# In pursuit of an HIV cure: from stem cell transplants to gene therapies

**DOI:** 10.3389/fgeed.2025.1634193

**Published:** 2025-09-05

**Authors:** Jennifer Clees, Maya Basic, Pedro E. Cruz, Servio H. Ramirez, Allison M. Andrews

**Affiliations:** Department of Pathology, Immunology and Laboratory Medicine, University of Florida, Gainesville, FL, United States

**Keywords:** HIV, hematopoietic stem cells, cure, bone marrow, gene therapy

## Abstract

Since 2009, seven people living with human immunodeficiency virus (PLHIV) have been declared cured of HIV after receiving allogeneic hematopoietic stem cell transplants (alloHSCTs) to treat hematologic malignancies. In this sense, cure signifies the absence of viral DNA/RNA and undetectable viral loads without the use of antiretroviral therapy (ART). Five of these transplants utilized mutated C-C motif chemokine receptor type 5 (CCR5^Δ32/Δ32^) stem cells. Much has been learned from these and past cases, and although effective, bone marrow transplants cannot be easily or safely translated to cure the millions of PLHIV across the globe. A successful eradicating cure includes both the prevention of HIV from entering new cells and the elimination of tissue reservoirs. Protecting hematopoietic stem and progenitor cells (HSPCs) from infection is a key consideration since there is evidence that HSPCs themselves, not only their descendants, are susceptible to infection. Gene therapy approaches have the potential to bring about an eradicating HIV cure that could be highly effective, broadly applicable, less expensive, and practical to implement. Current strategies are tackling this problem by removing the integrated proviral DNA from infected cells and/or eliminating the co-receptor(s) necessary for HIV viral entry into target cells. Both approaches hold promise, but they require overcoming key challenges (i.e., vector toxicity, transduction efficacy, elimination of reservoir cells, etc.). This review summarizes and examines the lessons learned about curing HIV through bone marrow transplants, the current gene therapy methodologies, pitfalls of eradication strategies as well as future directions of the field.

## 1 Introduction

Four decades after its identification as the causative agent of acquired immunodeficiency syndrome (AIDS), human immunodeficiency virus (HIV) continues to impact millions of people worldwide each year. In 2023, there were 39.9 million people living with HIV, including 1.3 million new cases (Global, 2024). In the US, HIV infections remain a significant public health challenge, affecting an estimated 1.2 million people with over 30,000 new infections annually (Author Anonymous, 2022). New HIV infections occur in an uneven distribution across the US, with southern states making up 49% of new HIV infections even though they account for only 38% of the population (Author Anonymous, 2018). HIV disproportionately burdens key marginalized populations. HIV cases in the US are concentrated in urban areas, where higher population density and more extensive transmission networks contribute to increased incidence rates (Pellowski et al., 2013).

HIV is managed with antiretroviral therapy (ART) to suppress the HIV viral load detected in a person’s blood. This reduction is achieved through consistent and correct use of ART, which prevents the virus from replicating effectively. Rarely, some individuals are able to naturally suppress the virus without the need for ART. These individuals are sometimes referred to as “elite controllers” (Navarrete-Muñoz et al., 2020). However, for the majority, lifelong treatment with ART is the only way to prevent viral rebound and disease progression. Adherence to treatment remains a challenge in the US nationwide and has been estimated to be between 60% and 90% (McComsey et al., 2021). Suboptimal ART levels allow the virus to develop mutations that can subsequently be more resistant to antiviral control (Aldous et al., 2017). Mutations in key viral enzymes, including reverse transcriptase, protease, and integrase, increase the virus’s genetic diversity and reduce ART effectiveness (Carr et al., 2023). Consequently, even with advances increasing the effectiveness of ART drugs, maintaining viral suppression is a lifelong effort that many fall short in sustaining.

When HIV infects cells, the viral ribonucleic acid (RNA) is reverse transcribed into deoxyribonucleic acid (DNA), which is then integrated into the host cell’s DNA. New infectious viral particles are created when the host cell machinery transcribes the DNA, with replication, assembly, and budding occurring. Resting or non-dividing cells, though, will maintain the integrated HIV and become a latent reservoir capable of passing the virus to progeny cells or, upon activation, spreading to uninfected cells. However, as active replication and virion production only occur at low levels with latently infected cells, ART cannot diminish the viral reservoir to fully eliminate HIV from these cells (Gupta et al., 2019; Renelt et al., 2022; Freen-van Heeren, 2022). If treatment is interrupted or drug resistance mutations occur, then the latent, replication-competent HIV reservoir can productively reseed the body (Allers et al., 2011).

To facilitate entry into host cells, HIV attaches to CD4 receptors and relies on the coreceptors CCR5 and CXCR4. Genetic mutations in the CCR5 coreceptor can influence both susceptibility to HIV infection and the progression of the disease. One notable mutation, known as CCR5-Δ32, results in a non-functional CCR5 receptor, which significantly reduces the virus’s ability to infect cells. Individuals who are homozygous for this mutation are largely or completely resistant to certain strains of HIV, particularly the common R5-tropic strain, providing a natural form of protection against the virus (de Silva and Stumpf, 2004). However, this mutation is relatively rare, occurring in about 1% of the European population and even less frequently in other populations (Solloch et al., 2017). Importantly, the mutation appears harmless to the individual and has thus spurred growing interest in gene therapy approaches aimed at combating HIV.

HIV displays features that effectively evade the immune system, and its biology has made it a formidable pathogen to treat and vaccinate against. The low fidelity function of the HIV reverse transcriptase enzyme makes it error-prone, meaning it frequently makes mistakes when copying the viral genome (Balzarini et al., 2001). As such, this in turn leads to rapid changes in HIV’s genetic material and proteins, including the envelope proteins (gp120 and gp41) that are targeted by the immune system. The mutations allow HIV to constantly alter its epitopes, which prevents antibodies from effectively neutralizing the virus and T-cells from recognizing and eliminating infected cells. The virus also downregulates MHC class I and II in infected cells, resulting in immune evasion from cytotoxic T lymphocytes (CTLs) that could identify and destroy infected cells. The targeting of CD4^+^ T-cells signifies the hallmark feature of HIV pathogenesis and the key aspect of how HIV evades the immune system. Specifically, the infection of CD4^+^ T-cells leads to their depletion, which critically weakens the immune system’s ability to combat the virus and increases susceptibility to opportunistic infections. Arguably one of the most challenging aspects for the body’s ability to combat HIV is its ability to establish latent reservoirs. These reservoirs in various tissues allow the virus to stay dormant and prevent detection by the immune system or accessibility for treatment. These reservoirs pose a significant challenge to achieving a cure for HIV. Other evasions and pathogenic mechanisms have been identified, including interference with innate immunity and the actions of HIV accessory proteins that can counteract antiviral enzymes and restriction factors. Since HIV is prone to a high rate of mutations that allows it to rapidly adapt and become resistant to a single medication, ART therapy utilizes the combination of multiple drugs to generate a higher barrier to resistance. ART helps to suppress viral replication more effectively by targeting multiple stages of the life cycle of HIV. Unfortunately, resistance can still develop while a person is on ART (known as virologic failure), and transmitted resistance can also occur in which individuals are infected with HIV strains that are already resistant to certain drugs.

The purpose of this mini review is to examine the history of curing HIV with bone marrow transplants, the susceptibility of stem cells to HIV, and the advances in gene therapies seeking to cure HIV in patients who may or may not have hematologic malignancies.

### 1.1 History of curing HIV with bone marrow transplants

#### 1.1.1 CCR5Δ32/Δ32 transplant cases

**FIGURE 1:**
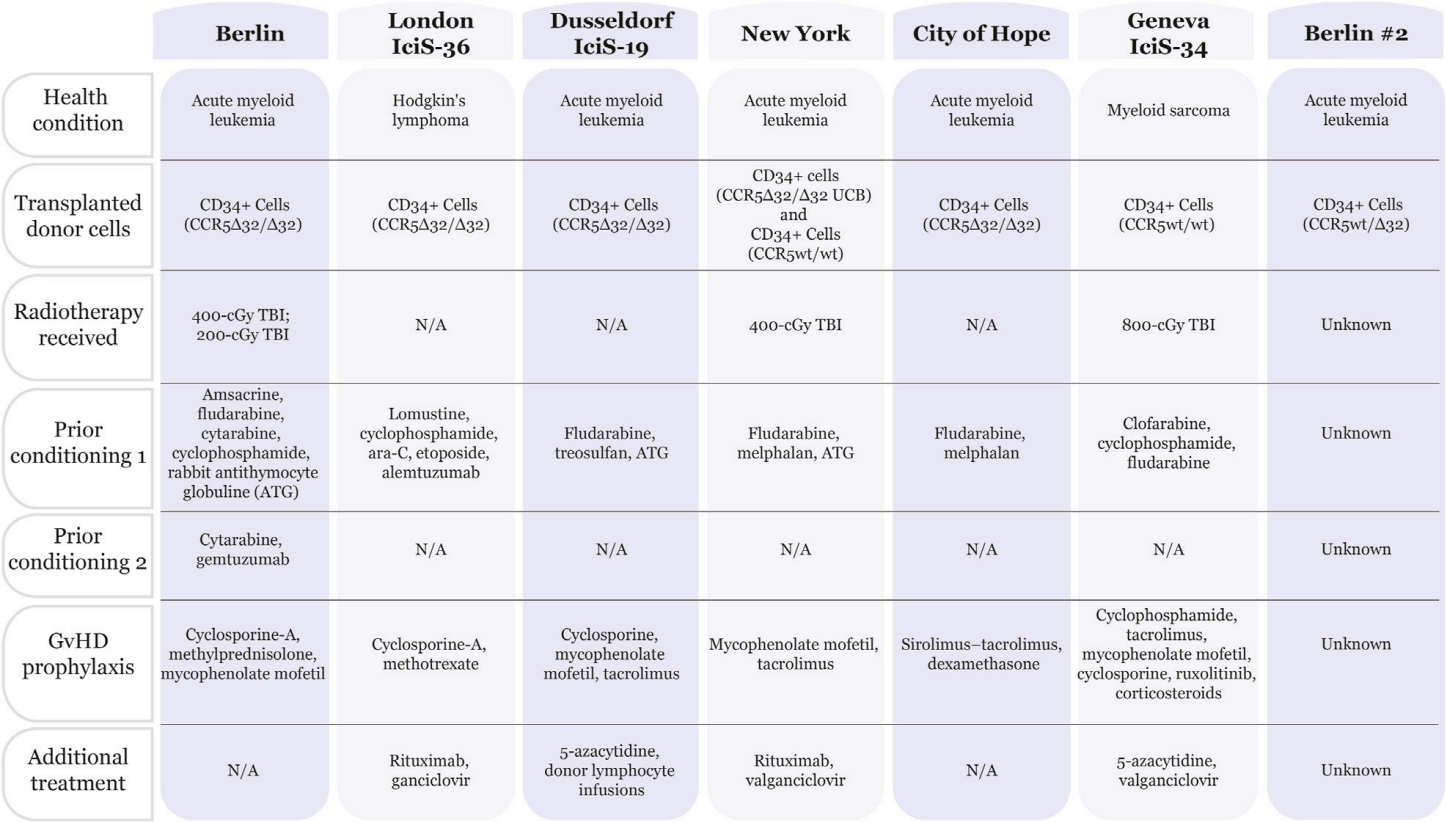
Chart summarizing patients that have been cured of HIV. Seven patients have been deemed “cured” from HIV infection. All patients were treated for cancer (acute myeloid leukemia, Hodgkin’s lymphoma or myeloid sarcoma) with bone marrow transplants. Five out of seven patients received CD34^+^ donor cells containing the CCR5Δ32/Δ32 mutation. In addition, the patients received a combination of treatments including pre-conditioning therapies and graft-versus-host disease (GvHD) prophylaxis, with or without radiotherapy. Summarized from (Hutter et al., 2009; Hsu et al., 2023; Dickter et al., 2024; Gaebler et al., 2024; Saez-Cirion et al., 2024; Gupta et al., 2019; Jensen et al., 2023).

To date, five people living with human immunodeficiency virus (PLHIV) have entered HIV remission as a result of their hematological cancer treatments with allogeneic hematopoietic stem cell transplants (alloHSCTs) from donors with homozygous 32 base-pair deletions in the C-C motif chemokine receptor type 5 (CCR5) allele (CCR5^Δ32/Δ32^) (Gupta et al., 2019; Allers et al., 2011; Hutter et al., 2009; Gupta et al., 2020; Jensen et al., 2023; Hsu et al., 2023; Dickter et al., 2024) (summarized in Figure 1). The deletions result in a frameshift mutation, causing the truncated protein to be nonfunctional and not expressed on the cell surface (Hsu et al., 2023). HIV type 1 (HIV-1) entry into host cells occurs via binding cluster of differentiation 4 (CD4) receptors along with co-receptors such as CCR5 or C-X-C motif chemokine receptor type 4 (CXCR4) (Freen-van Heeren, 2022; Hutter et al., 2009). HIV-1 may express tropism for CCR5 (R5-tropic), CXCR4 (X4-tropic), or both (dual-tropic). Tropism can be predicted by detecting the identity or change of a few amino acids in the V3 loop of the HIV-1 envelope (Env) protein, from more acidic/negatively charged in R5 strains to more basic/positively charged in X4 strains (Gupta et al., 2019; Renelt et al., 2022; Jensen et al., 2023). AlloHSCT from CCR5^Δ32/Δ32^ donors can result in complete donor chimerism, and thus, protection from infection with R5-tropic virions (Gupta et al., 2019; Hutter et al., 2009).

Of the five people in HIV remission following CCR5^Δ32/Δ32^ alloHSCT, four patients received donor CD34^+^ peripheral blood stem cells (PBSCs), and one patient received a haplo-cord transplant in which the PBSCs were CCR5^wt/wt^ and the umbilical cord blood unit (CBU) was CCR5^Δ32/Δ32^ (Gupta et al., 2019; Allers et al., 2011; Hutter et al., 2009; Gupta et al., 2020; Jensen et al., 2023; Hsu et al., 2023; Dickter et al., 2024). One patient also received donor lymphocyte infusions (DLI) to treat cancer relapse (Jensen et al., 2023). The transplants were treatment for acute myeloid leukemia (AML) in four cases and Hodgkin’s lymphoma (HL) in one case. Prior to transplantation, a reduced-intensity conditioning regimen without total-body irradiation (TBI) was used in three cases, while the other two patients received 4-Gy TBI. All patients received graft-versus-host disease (GvHD) prophylaxis. However, the haplo-cord transplant recipient was the only patient not to develop acute or chronic GvHD. Although each patient achieved complete donor chimerism, the samples used to reach this conclusion varied between peripheral blood and bone marrow. To determine whether HIV could rebound in these patients, analytical treatment interruption (ATI) occurred between 0 and 69 months post-alloHSCT. All the patients had been predominantly infected with R5-tropic HIV-1, and they entered HIV remission following transplantation. Numerous diagnostic methods and tissue samples were used to confirm remission, including verifying waning cellular and humoral immune responses to HIV. Although there were instances of positive detection for HIV-1 DNA, such as *env* and long terminal repeat (LTR), these trace amounts were considered ‘fossilized’ DNA and not indicative of the spread of replication-competent virus. All the patients exceeded 18 months of HIV remission, with the longest being 13 years (prior to death from AML relapse) (Gupta et al., 2019; Allers et al., 2011; Hutter et al., 2009; Gupta et al., 2020; Jensen et al., 2023; Hsu et al., 2023; Dickter et al., 2024).

#### 1.1.2 Interplay between graft-versus-host and graft-versus-reservoir effects

While many assume that protection against HIV infection is completely due to the CCR5^Δ32/Δ32^ alloHSCT transplant, the preconditioning treatment and ability of the graft to recognize the HIV reservoir as foreign (i.e., graft vs reservoir, GvR) play a role in the HIV cure (Gupta et al., 2019; Allers et al., 2011; Gupta et al., 2020; Jensen et al., 2023; Salgado et al., 2024). Homozygous CCR5 mutations may not be necessary given the recent declaration of a patient cured using a heterozygous CCR5^wt/Δ32^ alloHSCT (Gaebler et al., 2024). The patient has been virus free for over 5 years as of July 2024 without ART. However, the exact treatment details of this patient are currently unpublished. Additionally, a recent report detailed a patient receiving a CCR5^wt/wt^ alloHSCT (Geneva IciS-34) yet maintaining HIV-1 remission thus strongly supporting the contribution of additional aspects of the patient treatment in preventing viral rebound. The Geneva IciS-34 patient was diagnosed with myeloid sarcoma and received a conditioning regimen including 8-Gy TBI plus GvHD prophylaxis prior to transplant (Figure 1). ATI initiation 40 months post-alloHSCT showed HIV-1 remission in this patient for 32 months at the time of publication in 2024. Of note, the patient has been receiving near-continuous immunosuppressive treatment with ruxolitinib to treat acute and chronic GvHD. The authors point out that there is *in vitro* and *ex vivo* evidence for this JAK-STAT inhibitor preventing HIV from reactivating, replicating, and reseeding the viral reservoir (Saez-Cirion et al., 2024). Interestingly, a mathematical model using data from 30 patients predicts that reservoir depletion is independent of donor CCR5 status and instead depends on the conditioning chemotherapy and GvR effect, with the reservoir decay being proportional to T-cell chimerism (Salgado et al., 2024).

#### 1.1.3 Transplant cases involving viral rebound

While there are a number of cases in which patients have been deemed cured after years of evaluation during treatment interruption, several cases of viral rebound highlight the complexity in a sustainable cure. The most notable is the Essen patient who experienced a rapid rebound of a preexisting minority X4-tropic virus variant after CCR5^Δ32/Δ32^ alloHSCT(24). This case, for the first, demonstrated the weaknesses in the CCR5^Δ32/Δ32^ alloHSCT approach, which could not provide protection against viruses that use the CXCR4 receptor. These CXCR4 variants were determined to be from a tiny minority of viruses detected prior to transplantation. Analysis of samples from the Berlin patient, also showed evidence of X4-tropic viruses (Verheyen et al., 2019) which highlights the variability in reservoir reduction even among homologous transplants. Other cases of viral rebound further emphasize the incomplete reservoir decay from what was predicted using mathematical models. Patient IciS-28 showed rebound viremia 3 months after treatment interruption even though the patient’s HIV reservoir had been undetectable at 88 months post-CCR5^wt/wt^ alloHSCT (Salgado et al., 2024). Two Boston patients also experienced rebound viremia after CCR5^wt/wt^ alloHSCT despite high levels of chimerism in the peripheral blood and the virus being undetectable prior to ATI (Henrich et al., 2014). The length of undetectable virus in Patient IciS-28 points to a latent reservoir that remains dormant for long periods of time before reactivating and reinfecting the entire immune system. This story is similar to the Mississippi baby who was treated aggressively with ART at birth and had undetectable virus without ART for 27 months before viral rebound from a latent reservoir (Ledford, 2014). Consequently, due to the difficulty in fully depleting the HIV reservoir, strategies that can prevent cells from being reinfected are critical to clearing the virus. Therefore, CCR5 status remains a critical determinant for HIV remission after transplantation, as repopulated daughter cells are not susceptible to R5-tropic HIV.

### 1.2 Can HIV infect HSCs?

In the context of these successes and failures in curing HIV, a question emerges–are the hematopoietic stem and progenitor cells (HSPCs) themselves susceptible to infection with HIV-1 or just their differentiated progeny, e.g., macrophages, dendritic cells (DCs), and CD4^+^ T-cells? The importance of this distinction lies in the essential function the viral reservoir plays in HIV escaping immune detection and pharmaceutical intervention to allow for further proliferation. Viral persistence despite ART is achieved via latency, in which HIV only replicates its genetic material or assembles new virions at low levels but is readily reactivated from the reservoir of integrated proviral genomes. Memory CD4^+^ T-cells notoriously contribute to the reservoir but are not the sole supplier of HIV-1 to uninfected cells. There is *in vitro* evidence using staining and flow cytometry that HSCs, multipotent progenitors (MPPs), and lineage-committed progenitors, i.e., common myeloid progenitors (CMPs) and common lymphoid progenitors (CLPs), can not only co-express CD4 and CXCR4 or CCR5 but also can be infected with HIV-1. Moreover, re-plating assays demonstrate that these cells maintain the capacity for multi-lineage differentiation. In addition, the genomic DNA of CD34^+^ cells from bone marrow biopsy samples of 11 PLHIV on ART tested positive on qPCR for HIV-1 *gag* DNA in eight cases, but the authors could not show consistent evidence of integrated proviral DNA in these samples due to the limited number of CD34^+^ cells available for sequencing. These *in vitro* and *in vivo* results indicate that HSPCs can contribute to the viral reservoir. *In vitro* experiments also demonstrated a preferential infection of HSPCs double-positive for CD4 and CXCR4 over CD4 and CCR5. However, the frequency of the CD4/CXCR4 double-positive cells was ∼4–5%, as compared to less than 1% for CD4/CCR5 HSCs from cord blood and bone marrow (Renelt et al., 2022; Karuppusamy et al., 2022). Although this is a small population, the large number of progenies that can be produced would thus increase the frequency of infected daughter cells in circulation. A remaining question is whether there are conditions that increase the expression of the HIV receptors/co-receptors in CD34^+^ cells, which would augment the susceptibility to HIV. Additionally, studies that estimated the prevalence of HSPCs that are susceptible to HIV were limited to a handful of donors and may not capture whether any population differences in receptor expression exist. Comprehensive studies that evaluate the prevalence of HSPC susceptibility to HIV infection are therefore needed. Particularly, in regard to varied physiological conditions such as the role of comorbidities and inflammatory status and demographics (as a function of sex, age, ethnicity, etc.) that could also affect HSPC response to HIV infection. An understanding of HIV infection on HSPC could lead to important insights about pathogenesis and curative approaches.

### 1.3 Using gene therapy to cure HIV

With bone marrow transplantation being limited in its use as a cure, there is great interest in gene therapy strategies to eliminate HIV (summarized in Table 1). Thus far, successful cell targets for receptor gene editing have included primary human CD4^+^ T-cells, T-cell and macrophage cell lines, adipose stem cells (ASCs), induced pluripotent stem cells (iPSCs), and HSCs. However, a key benefit to targeting cells capable of hematopoiesis means that daughter cells can inherit the mutation, with the goal being complete repopulation of cells with resistance to HIV infection (Freen-van Heeren, 2022; Psatha et al., 2022). Although there is evidence for knocking out CCR5, CXCR4, or both receptors *in vitro*, and the field of gene therapy has taken leaps from *ex vivo* to *in vivo* therapeutics for other conditions, there is not yet clinical trial evidence for achieving high percentages of CCR5, CXCR4, or HIV knockout (KO) in humans (Freen-van Heeren, 2022; Psatha et al., 2022). The remaining body of the review will summarize the strides taken thus far, the failures, and the challenges to overcome.

**TABLE 1:** Studies for which a gene therapy modality has been used to neutralize, excise, or eliminate HIV. The table includes subcategories starting with known gene therapy methods to counter HIV in a clinical setting. The next set of subcategories are divided into whether the delivery of the modifying genetic cargo to cells features AAV (the most commonly used viral construct in gene therapy applications) or non-AAV approaches in preclinical models.

HIV clinical trials using gene therapy				
Study/Year	Gene targeting or eradication strategy	Target gene	Delivery method	Cell type or model organism
NCT02388594, NCT02225665 NCT04201782, (Tebas et al., 2021)	ZFN	CCR5	mRNA	Modified Autologous T-cells infused back into the patient
NCT03617198	ZFN and CAR-T	CCR5	mRNA	Modified Autologous T-cells infused back into the patient
NCT02500849	ZFN	CCR5	Transfection	Modified autologous CD34^+^ Cells infused back into the patient
NCT01252641, NCT01044654, NCT01543152, NCT03666871, NCT04201782, NCT00842634 (Tebas et al., 2014)	ZFN	CCR5	Adenovirus	Modified autologous T-cells infused back into the patient
NCT01787994, (Jacobson et al., 2021)	MazF-T	CCR5	Lentivirus	Modified autologous T-cells infused back into the patient
NCT01734850	shRNA	CCR5, C46	LVsh5/C46	Modified autologous T-cells and HSPCs infused back into the patient
NCT03215004, (Muvarak et al., 2022)	RNAi	CCR5, Tat, Vif	Lentivirus	Modified autologous T-cells infused back into the patient
NCT03164135, (Xu et al., 2019)	CRISPR/Cas9	CCR5	Transfection	Modified allogenic HSPCs infused into the patient
NCT05144386	CRISPR/Cas9	LTR, Gag	AAV9	Intravenous administration of the viral vector
NCT01937455, (Priddy et al., 2019)	bNAb	PG9	AAV1	Intramuscular administration of the viral vector
NCT03374202, (Casazza et al., 2022)	bNAb	VRC07	AAV8	Intramuscular administration of the viral vector

AAV mediated gene editing				
Study/Year	Gene targeting or eradication strategy	Target gene	Delivery method	Cell type or model organism
Yin et al. (2017)	CRISPR/Cas9	HIV LTR, Gag	AAV-DJ8	Humanized mice
Kunze et al. (2018)	CRISPR/Cas9	HIV LTR	AAV9P1	*In-vitro* (Astrocytes)
Dash et al. (2019)	CRISPR/Cas9	HIV LTR, Gag	AAV9	Humanized mice
Theuerkauf et al. (2023)	CRISPR/Cas9	HIV LTR	AAV2-DARPins	Humanized mice
Burdo et al. (2024)	CRISPR/Cas9	HIV LTR, Gag	AAV9	Rhesus macaques
Dudek et al. (2024)	CRISPR/Cas9	CCR5, CXCR4	AAV6	Humanized mice
Feist et al. (2025)	CRISPR/Cas9	CCR5	AAV6	Humanized mice

AAV mediated bNAb expression				
Study/Year	Gene targeting or eradication strategy	Target gene	Delivery method	Cell type or model organism
Lewis et al. (2002)	bNAb	b12	AAV2	RAG-1 Mice
Johnson et al. (2009)	bNAb	4L6, 5L7, N4 immunoadhesins	AAV1	Rhesus macaques
Balazs et al. (2011)	bNAb	VRC01, b12, 2G12, 4E10, 2F5	AAV8	HuPBMC-NSG humanized mice, Rag2/γc (RAG), B6, and Balb/C
Horwitz et al. (2013)	bNAb	10-1074, 3BNC117	AAV8	Humanized mice- HuPBMC
Balazs et al. (2014)	bNAb	VRC01, b12, VRC07	AAV8	Humanized mice, NSG and BLT
Gardner et al. (2015)	bNAb	eCD4-Ig	AAV1	Rhesus macaques
Saunders et al. (2015)	bNAb	VRC01	AAV8	Rhesus macaques
Fuchs et al. (2015)	bNAb	4L6, 5L7	AAV1	Rhesus macaques
Martinez-Navio et al. (2016)	bNAb	4L6, 5L7, 3BNC117, 10E8, 10-1074, 1NC9, 8ANC195	AAV1	Rhesus macaques
Badamchi-Zadeh et al. (2018)	bNAb	PGT121	AAV1, AAV5	Humanized Mice BLT mice, BALB/c, Rag KO mice
Welles et al. (2018)	bNAb	ITS01, ITS06.02, ITS11, ITS08, ITS10	AAV8	Rhesus macaques
van den Berg et al. (2019)	bNAb	CAP256-VRC26.25	AAV2, AAV8	NMRI mice
Gardner et al. (2019b)	bNAb	3BNC117, NIH45-46, 10-1074, PGT121	AAV1	Humanized mice, Rhesus macques
Shipulin et al. (2024)	bNAb	N6, 10E8, 10-1074, VRC07-523, PGDM1400, 10-1074	AAV9	CBAxC57Bl, C57BL/6
Martinez-Navio et al. (2019)	bNAb	3BNC117, 10-1074, N6, PGT128, PGT145, 35o22	AAV1	Rhesus macaques
Fuchs et al. (2020)	bNAb	4L6	AAV1, AAV8	Rhesus macaques
Huang et al. (2020)	bNAb	VRC01	AAV2	C57Bl/6J
Nahmad et al. (2022)	bNAb	3BNC117	AAV-DJ	CD45.2 C57BL/6OlaHsd (Envigo) mice
Davis-Gardner et al. (2023)	bNAb	ITS01	AAV1, AAV8, AAV9, AAV-NP22 or AAV-KP1	Rhesus macaques

AAV other				
Study/Year	Gene targeting or eradication strategy	Target gene	Delivery method	Cell type or model organism
Horster et al. (1999)	HIV antisense/ribozyme	HIV-1-directed antisense sequence AR6, hammerhead ribozyme 2as-Rz12	AAV2	HeLaP4/CCR5, T-lymphocytes, macrophages, CD34^+^ cells
Boden et al. (2003)	RNA inhibitors	Tat	AAV2	H9 cells
Sather et al. (2015)	TALE DNA binding domain + sequence-specific HE	CCR5	AAV6	T-cells, CD34^+^ peripheral blood mononuclear cells
Gardner et al. (2019a)	HIV entry inhibitor	eCD4-Ig	AAV1	Rhesus macaques
Tang et al. (2024)	HIV latency reactivator	exosomal Tat	AAV-DJ	Balb/cJ

Non-AAV gene therapy related approaches without the use of CRISPR/Cas9				
Study/Year	Gene targeting or eradication strategy	Target gene	Delivery method	Cell type or model organism
Perez et al. (2008)	ZFN	CCR5	Adenovirus Ad5/35	T-cells, NOG mice
Schleifman et al. (2011)		CCR5	Transfection	CD34^+^ cells
Didigu et al. (2014)	ZFN	CCR5, CXCR4	Adenovirus Ad5/F35	T-cells, humanized mice
Ru et al. (2013)	TALEN	CCR5	Cell permeable	Hela, iPSCs
Qu et al. (2013)	ZFN	LTR	Transfection	Jurkat, T-cells
Hofer et al. (2013)	ZFN	CCR5	Transfection	Humanized mice
Li et al. (2013)	ZFN	CCR5	Adenovirus (Ad5/35)	CD34^+^ HSPCs, humanized mice
McNeer et al. (2013)	PNAs	CCR5	poly (lactic-co-glycolic acid) (PLGA) nanoparticles	NSG mice
Schleifman et al. (2013)	PNAs	CCR5	poly (lactic-co-glycolic acid) (PLGA) nanoparticles	huPBMC NSG mice
Badia et al. (2014)	ZFN	CCR5	Transfection	TZM-bl cells
Yi et al. (2014)	ZFN	CCR5	Lentivirus (LV)	Humanized mice
Wolstein et al. (2014)	shRNA	CCR5, C46	Lentivirus (LV)	T-cells, HSPCs, Molt4/CCR5 and CEM.NKR.CCR5
Mock et al. (2014)	TALEN	CCR5	Lentivirus (LV)	CCR5+-GHOST cell line Jurkat cells
Liu et al. (2014)	TALEN	CCR5, BMPR1A	Transfection	HeLa, HEK293T
Fadel et al. (2014)	TALEN	PSIP1	Lentivirus (LV)	HEK293T, Jurkat
Shimizu et al. (2015)	RNAi	CCR5	Lentivirus (LV)	BLT humanized mice
Choi et al. (2015)	shRNA	CCR5, Gag, Env, Tat, Pol, Vif	Lentivirus (LV)	T-cells, Hu-PBL mice
Strong et al. (2015)	TALEN	transactivation response element	Transfection	HeLa-tat-III/LTR/d1EGFP cells
Manotham et al. (2015)	ZFN	CCR5	Transfection	Mesenchymal stem cells
Mock et al. (2015)	TALEN	CCR5	Transfection	T-cells
Peterson et al. (2016)	ZFN	CCR5	Electroporation	CD34^+^ HSPCs, macaques
Peterson et al. (2017)	ZFN	CCR5	Electroporation	CD34^+^ HSPCs, macaques
Peterson et al. (2018)	ZFN	CCR5	Transfection	CD34^+^ HSPCs, macaques
Chattong et al. (2018)	ZFN	CCR5	Transfection	CD34^+^ cells
Nerys-Junior et al. (2018)	TALEN	CCR5	Transfection	HEK293T
Okee et al. (2018)	ZFN	pol	Transfection Adenovirus (Ad5)	TZM-bl and ACH-2/J-Lat cells
Ji et al. (2018)	ZFN	LTR	Transfection	T-cells, C11 cells
Liu et al. (2018)	ZFN	CCR5	Transfection	HeLa, HEK293T
Romito et al. (2021)	TALEN	CCR5	Transfection	T-cells
Card et al. (2021)	ZFN	CCR5	Electroporation	macaques
Knipping et al. (2022)	BE	CCR5	Transfection	CD34^+^ HSPCs
DiGiusto et al. (2016)	ZFN	CCR5	Transfection	CD34^+^ HSPCs
Li et al. (2020)	RNAi	CCR5, Tat, Vif	Lentivirus (LV)	T-cells

Non-AAV gene therapy related approaches that uses CRISPR/Cas9				
Study/Year	Gene targeting or eradication strategy	Target gene	Delivery method	Cell type or model organism
Cradick et al. (2013)	CRISPR/Cas9	CCR5	Transfection	HEK293
Ebina et al. (2013)	CRISPR/Cas9	LTR	Lentivirus (LV)	Jurkat
Hu et al. (2014)	CRISPR/Cas9	LTR	Transfection	CHME5 microglial cells, U1 cells, TZM-bl cells
Liao et al. (2015)	CRISPR/Cas9	LTR	Lentivirus (LV)	Sup-T1 cells, hPSC-differentiated monocytes/macrophages
Li et al. (2015)	CRISPR/Cas9	CCR5	Adenovirus	T-cells
Kang et al. (2015)	CRISPR/Cas9	CCR5	Transfection	iPSCs
Zhu et al. (2015)	CRISPR/Cas9	LTR, pol, tat/rev	Lentivirus (LV)	J-Lat10.6
Hou et al. (2015)	CRISPR/Cas9	CXCR4	Lentivirus (LV)	T-cells
Wang et al. (2016a)	CRISPR/Cas9	LTR, HIV-1 sense and antisense strand, protein-encoding sequences	Lentivirus (LV)	HEK293T, SupT1 T cells
Wang et al. (2016c)	CRISPR/Cas9	LTR	Lentivirus (LV)	HEk293T, SupT1, TZM-bl
Wang et al. (2016b)	CRISPR/Cas9	LTR, 5′and 3′ends, nef, gag, pol, TatRev, Env	Lentivirus (LV)	SupT1 cells
Kaminski et al. (2016a)	CRISPR/Cas9	LTR	Lentivirus (LV)	PBMC
Kaminski et al. (2016b)	CRISPR/Cas9	LTR	Lentivirus (LV)	Jurkat, 2D10, TZM-bl cells
Yin et al. (2016)	CRISPR/Cas9	LTR	Lentivirus (LV)	HEK293T
Lebbink et al. (2017)	CRISPR/Cas9	LTR, matrix, protease, reverse transcriptase, integrase	Lentivirus (LV)	Sup-T1 cells, J-Lat
Ehrke-Schulz et al. (2017)	CRISPR/Cas9	CCR5	Adenovirus	A549
Xu et al. (2017)	CRISPR/Cas9	CCR5	Transfection	Humanized mice
Seki and Rutz (2018)	CRISPR/Cas9	CXCR4, CD127, and CCR7	Transfection	Mouse and human T-cells
Liu et al. (2018)	CRISPR/Cas9	CCR5	Transfection	HeLa, HEK293T
Nerys-Junior et al. (2018)	CRISPR/Cas9	CCR5	Transfection	HEK293T
Ophinni et al. (2018)	CRISPR/Cas9	Tat, Rev	Lentivirus (LV)	HEK293T, Hela, L2 cells J-Lat cells
Wang et al. (2018b)	CRISPR/Cas9	primer binding site	Lentivirus (LV)	Hek293T, TZM-bl cells SupT1 cells
Okee et al. (2018)	CRISPR/Cas9	pol	Transfection	TZM-bl and ACH-2/J-Lat cells
Yin et al. (2020)	CRISPR/Cas13a	LTR, gag, tat, rev	Lentivirus (LV)	HEK293T, Jurkat, JLAT10.6
Gao et al. (2020)	CRISPR/Cas12a	sense and antisense, LTR	Lentivirus (LV)	HEK293, SupT1 cells
Ophinni et al. (2020)	CRISPR/Cas9	Tat, rev	Lentivirus (LV)	MT-4 T cells
Schmidt et al. (2020)	CRISPR/Cas9	CCR5	microinjection pipette	Macaque embryos
Nguyen et al. (2021)	CRISPR/Cas13d	Gag, pol, cPPT	Lentivirus (LV)	HEK293, J1.1, TXM-bl, T-cells
Herskovitz et al. (2021)	CRISPR/Cas9	Tat/Rev/Env	Transfection, lentivirus (LV), lipid nanoparticle	ACH2 T cells, J-Lat, U1 cells
Lai et al. (2021)	CRISPR/Cas9	LTR	Transfection	HEK293T, Jurkat
Fan et al. (2022)	CRISPR/Cas12a	LTR, Gag, Tat, Tat/rev	Lentivirus (LV)	SupT1 cells
Li et al. (2022a)	CRISPR/Cas9	CCR5, CXCR4	Lentivirus (LV)	Humanized mice
Claiborne et al. (2025)	CRISPR/Cas9	CCR5	Transfection	Humanized mice

Abbreviations: BE, base editors; bNAb, broadly neutralizing antibodies; CAR-T, chimeric antigen receptor T; MazF-T, MazF-modified CD4 + T; RNAi, RNA interference; shRNA, short hairpin RNA; TALENs, transcription activator-like Effector nucleases; PNAs, Triplex-forming peptide nucleic acids; ZFN, zinc finger nucleases; HE, homing endonuclease. (Fuchs et al., 2020; Gao et al., 2020; Gardner et al., 2019a; Gardner et al., 2019b; Gardner et al., 2015; Herskovitz et al., 2021; Hofer et al., 2013; Horster et al., 1999; Horwitz et al., 2013; Hou et al., 2015; Hu et al., 2014; Huang et al., 2020; Jacobson et al., 2021; Ji et al., 2018; Johnson et al., 2009; Kaminski et al., 2016a; Kaminski et al., 2016b; Kang et al., 2015; Kunze et al., 2018; Lai et al., 2021; Lebbink et al., 2017; Lewis et al., 2002; Li et al., 2015; Li et al., 2020; Li et al., 2013; Liao et al., 2015; Liu et al., 2014; Liu et al., 2018; Manotham et al., 2015; Martinez-Navio et al., 2019; Martinez-Navio et al., 2016; McNeer et al., 2013; Mock et al., 2015; Mock et al., 2014; Muvarak et al., 2022; Nahmad et al., 2022; Nerys-Junior et al., 2018; Nguyen et al., 2021; Okee et al., 2018; Ophinni et al., 2018; Ophinni et al., 2020; Perez et al., 2008; Peterson et al., 2017; Peterson et al., 2018; Priddy et al., 2019; Qu et al., 2013; Romito et al., 2021; Ru et al., 2013; Sather et al., 2015; Saunders et al., 2015; Schleifman et al., 2011; Schleifman et al., 2013; Schmidt et al., 2020; Seki and Rutz, 2018; Shimizu et al., 2015; Shipulin et al., 2024; Strong et al., 2015; Tang et al., 2024; Tebas et al., 2021; Tebas et al., 2014; Theuerkauf et al., 2023; van den Berg et al., 2019; Wang et al., 2016a; Wang et al., 2016b; Wang Z. et al., 2016; Wang Z. et al., 2018; Welles et al., 2018; Wolstein et al., 2014; Xu et al., 2017; Yi et al., 2014; Yin et al., 2016; Yin et al., 2017; Zhu et al., 2015).

Earlier gene editing strategies, such as zinc-finger nucleases (ZFNs) and transcription activator-like effector nucleases (TALENs), have been more recently replaced with the clustered regularly interspaced short palindromic repeats (CRISPR)-associated nuclease Cas9 (CRISPR-Cas9) system (Psatha et al., 2022). In the earliest study of CCR5 KO in nonhuman primates (NHPs), one group demonstrated the successful engraftment of CD34^+^ cells with CCR5 disrupted by ZFNs, but in the months following the transplant, the percentage of CCR5-disrupted progeny cells in the peripheral blood was 3%–5% (Psatha et al., 2022; Peterson et al., 2016). In one clinical trial (NCT03164135), a patient was successfully treated for acute lymphoid leukemia (ALL) with a CRISPR-Cas9 CCR5-ablated HSPC transplant, achieving engraftment and remission, but the patient’s HIV was not cured, with the percent of lymphocytes maintaining the CCR5 KO being ∼5% and HIV rebounding after ATI (Freen-van Heeren, 2022; Xu et al., 2019). In addition, there have been efforts to move away from strategies that rely upon double-stranded breaks (DSBs) repaired by endogenous DNA repair mechanisms such as non-homologous end joining (NHEJ), an error-prone process intentionally leading to insertions and deletions (indels) in the target gene sequence and potential p53 DNA damage response activation, to strategies that allow more precise point mutations, such as base editors (BEs) and prime editors (PEs) (Psatha et al., 2022). While PEs have not yet been used in the context of HIV, adenine BEs have been used to KO CCR5 in HSPCs (Psatha et al., 2022; Knipping et al., 2022). CXCR4 KO is less well-studied, with the receptor playing a role in HSPC migration. There has been some work to study dual (CCR5 and CXCR4) KO via CRISPR-Cas9 in primary CD4^+^ T-cells and murine models. While high HIV-1 resistance occurred *in vitro*, engraftment was poor *in vivo*, which may be due to disruption of CXCR4, which altered engraftment and homing to the bone marrow (Freen-van Heeren, 2022; Li S. et al., 2022).

Beyond co-receptor targeting, there has been some work to excise the proviral HIV reservoir, although this approach would not be protective against future infection. In 2024, Excision BioTherapeutics’ EBT-101 Phase I/II clinical trial (NCT05144386) used an AAV9 vector for *in vivo* gene therapy delivering CRISPR-Cas9 and two gRNAs targeting three sites on the HIV proviral DNA and proved safe in humans. Of the five patients in this trial, only three patients underwent ATI, with one patient maintaining viral suppression for 4 months post-ATI while the other two rebounded immediately. Excision BioTherapeutics is now working to test a higher dose and explore other delivery methods, such as lipid nanoparticles (LNPs), in order to prevent viral rebound post-ATI in its clinical trial participants and future patients (Highleyman, 2024). Another group targeted HIV-1 RNA with CRISPR-Cas13 in HEK293T cells, not only successfully reducing the level of viral RNA produced from transfected plasmid and proviral DNA, but also HIV-1 RNA from virions entering the cells. However, the researchers did not eliminate all viral RNA, and this approach does not destroy the HIV DNA present within infected cells (Psatha et al., 2022; Yin et al., 2020). Work with CRISPR-Cas13 in primary CD4^+^ T-cells and HSCs could further support this approach to targeting HIV-1 RNA directly.

Viral vector delivery of gene therapies does pose some challenges in terms of immune response-induced reduction of transduction efficiency. For example, in lentiviral (LV) vectors, the host immune system may respond to the packaging cell major histocompatibility complexes (MHCs) on the virus envelope surface, resulting in antibody‐dependent complement‐mediated inactivation and antigen presentation to T-cells. In adeno-associated viral (AAV) vectors, the kilobase (kb) packaging limit is rather low, and serum neutralization may occur, but increasing the dose to overcome transduction inhibition has caused hepatotoxicity in clinical trials. Seven deaths are known to be associated with acute liver failure occurring during treatment with AAV-based gene therapies. Adenoviral (Ad) vectors also must navigate the host immune response and potential cytokine storm, but helper-dependent hybrid Ad5/35 (HdAd5/35) has been used with a transposon-based approach to accomplish *in vivo* HSC gene editing (Psatha et al., 2022; Wang H. et al., 2018). In order to sidestep considerations of host immune responses to viral vectors, chemical means, such as nanoparticle delivery systems, are also being investigated (Psatha et al., 2022). Organic nanoparticles, more specifically LNPs, have been used extensively to deliver mRNA-based vaccines against SARS-CoV-2. Moreover, with bone marrow being a possible target site for gene therapy to cure HIV and its reservoir, one must consider the difficulties in targeting and transducing various HSC populations within this complex niche with intravenous (IV) administration, such as loss to/uptake in highly vascular tissues. Consequently, HSC mobilization and *ex vivo* strategies may be used to improve outcomes. Intraosseous (IO) administration may also be a potential strategy, but this invasive approach does not result in uniform administration to all bone marrow sites, and with aging, the bone marrow composition changes and fat replaces the largest and perhaps more accessible sites for hematopoiesis (i.e., femurs). Thus, the transduction efficiency achievable with the IO approach is questionable, particularly in the case of an HIV cure in which the goal is to eliminate the viral reservoir (Psatha et al., 2022).

### 1.4 Future directions

While this mini review has highlighted the transplant and gene therapy pursued by researchers, other therapeutic avenues are also being considered. The latent reservoir remains a major challenge that allows reseeding of the virus after therapy. There are numerous tissue reservoirs of HIV, such as gut-associated lymphoid tissue (GALT) and the central nervous system (CNS), that persist despite ART (Boesecke, 2023). One strategy to eliminate the reservoir involves latency-reversing agents (LRAs) to “shock” the integrated provirus into activating and replicating, then “kill” the infected cells via the host immune system, while continuing ART to prevent HIV proliferation to previously uninfected cells (Renelt et al., 2022). Therefore, it is a point of curiosity: if LRAs were used in combination with gene therapies, would the results recapitulate the successes of conditioning plus CCR5^Δ32/Δ32^ alloHSCTs? Moreover, could the conditioning regimen (chemotherapy drugs and potentially radiation) and/or immunosuppressive drugs to prevent or treat GvHD, such as ruxolitinib, be repurposed and coupled with gene therapies? The conditioning regimen might contribute to reservoir reduction, while the receptor(s) or virus are subject to KO with gene therapy. Likewise, immunosuppressive drugs might help prevent reseeding or suppress immune responses to viral or nonviral vectors while patients are treated with gene therapies. One group using HdAd5/35 to treat hemoglobinopathies in NHPs evaluated a dexamethasone, anti-interleukin (IL)-1, and anti-IL-6 pretreatment resulting in limited host immune responses. Still, this approach has not yet been tested in the context of gene therapies for HIV (Psatha et al., 2022; Li C. et al., 2022). Beyond these questions, there are also opportunities to improve gene therapy candidates and increase transduction efficiencies while maintaining patient safety. As noted previously, there is not yet clinical trial evidence for high percentages of co-receptor or HIV KO with gene therapies, so further optimization is necessary.

## 2 Conclusion

Since the beginning of the HIV/AIDS pandemic, tens of millions of people have lost their lives to AIDS-related illnesses and over one million people were newly infected with HIV in 2023(1). While ART has drastically altered the landscape, enabling PLHIV to achieve undetectable viral loads and prevent further transmission, a cure that eliminates the necessity of lifelong drug therapy for all PLHIV remains elusive. Although seven cases using alloHSCT to cure HIV have been reported to date, five of which involved transplants with the rare CCR5^Δ32/Δ32^ mutation, such an invasive procedure requiring physicians with expertise in bone marrow transplantation cannot be readily translated globally to millions of people. Furthermore, as we learn more from these unique cases where HIV has been cured, only time will tell whether they encompass a sterilizing cure, in which HIV has been completely eradicated in the body or a functional remission, where long-term control of HIV replication and transmission without ART is accomplished even though virus may still be present in the body. Regardless, bone marrow transplants for HIV treatment are not scalable solutions due to the highly invasive and intensive nature of the procedures. Eliminating a patient’s immune system with chemotherapy or radiation before transplanting healthy stem cells comes with many risks. This process can lead to serious and potentially life-threatening complications like severe infections and graft-versus-host disease. The procedure is considered costly and requires significant resources, making it impractical for the vast majority of people living with HIV worldwide. Importantly, finding suitable donors with the specific genetic criteria needed for a successful transplant, particularly those with natural resistance to HIV infection, is a significant challenge. However, the knowledge gained from these case reports has opened avenues of research into using gene therapy to remove the co-receptors necessary for viral entry or remove the virus itself from infected cells. Current work with CRISPR-based gene therapies in clinical trials shows promise for the future, but less invasive approaches to an HIV cure would likely be required to expand their reach to the millions of PLHIV globally.
